# Loss of *Dictyostelium *HSPC300 causes a *scar*-like phenotype and loss of SCAR protein

**DOI:** 10.1186/1471-2121-10-13

**Published:** 2009-02-19

**Authors:** Alice Y Pollitt, Robert H Insall

**Affiliations:** 1 School of Biosciences, University of Birmingham, Birmingham, B15 2TT, UK; 2 Beatson Institute for Cancer Research, Switchback Road, Bearsden, Glasgow, G61 1BD, UK; 3 School of Medicine, University of Birmingham, Birmingham, B15 2TT, UK

## Abstract

**Background:**

SCAR/WAVE proteins couple signalling to actin polymerization, and are thus fundamental to the formation of pseudopods and lamellipods. They are controlled as part of a five-membered complex that includes the tiny HSPC300 protein. It is not known why SCAR/WAVE is found in such a large assembly, but in *Dictyostelium *the four larger subunits have different, clearly delineated functions.

**Results:**

We have generated *Dictyostelium *mutants in which the HSPC300 gene is disrupted. As has been seen in other regulatory complex mutants, SCAR is lost in these cells, apparently by a post-translational mechanism, though PIR121 levels do not change. HSPC300 knockouts resemble *scar *mutants in slow migration, roundness, and lack of large pseudopods. However *hspc300*-colonies on bacteria are larger and more similar to wild type, suggesting that some SCAR function can survive without HSPC300. We find no evidence for functions of HSPC300 outside the SCAR complex.

**Conclusion:**

HSPC300 is essential for most SCAR complex functions. The phenotype of HSPC300 knockouts is most similar to mutants in *scar*, not the other members of the SCAR complex, suggesting that HSPC300 acts most directly on SCAR itself.

## Background

The WASP/SCAR family of proteins are key regulators of actin polymerisation, connecting signalling molecules to the activation of the Arp2/3 complex. SCAR/WAVE proteins, in particular, play an important role in the regulation of actin dynamics at the leading edges of moving cells. Biochemical studies in a range of organisms demonstrate that SCAR/WAVE is found in a 1:1:1:1:1 complex with four other proteins (PIR121, Nap1, Abi2 and HSPC300) [1,2]. It is becoming clear, in particular from studies in *Dictyostelium*, that individual components of the complex regulate SCAR through different signalling pathways and that some may also have additional SCAR independent functions *in vivo*. [3-6] Most evidence now suggests that all members of the complex are needed for the correct localisation and function of SCAR [7,8].

The smallest SCAR complex member, HSPC300, ranges from 68 to 110 amino acids in length, giving a size of between 8 and 14 kDa. Surprisingly little is known about its contribution to SCAR complex function and stability, perhaps because of the experimental difficulty associated with its smallness. Several studies investigating the function of the plant HSPC300 homologue, BRICK1, have found that is plays a crucial role in cytoskeletal remodelling. Maize BRICK1 null mutations lead to defects in the localisation of cortical actin in dividing and expanding leaf epidermal cells. These cells fail to undergo specific shape changes in preparation for asymmetric cell division [9]. *Arabidopsis *HSPC300 has been shown to be needed for SCAR complex stability, yet others have demonstrated *in vitro *that HSPC300 is not fundamental for the formation of the complex [1,10]. HSPC300 has also been shown to be important in the *Drosophila *nervous system, in which disruption of HSPC300 leads to a similar phenotype seen in other SCAR complex mutants [11].

In this work we characterise the *Dictyostelium hspc300 *gene, and assess the consequences of its disruption.

## Results and discussion

### *Dictyostelium *HSPC300

**Figure 1:**
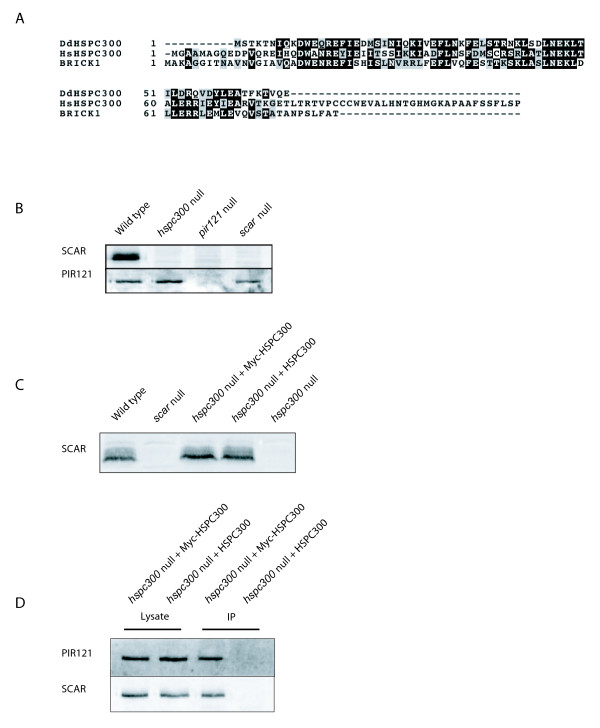
**HSPC300 as part of the SCAR complex**. **(A) **Alignment of HsHSPC300 (Accession number AAF28978), AtHSPC300 (Accession number Q94JY4) and DdHSPC300 (Dictybase reference DDB0231424). **(B) **Western blots showing SCAR and PIR121 levels in growing cells. Equal quantities of protein were probed with anti-SCAR and PIR121 antibodies. SCAR protein is almost undetectable in IR55 *hspc300 *null cells. PIR121 levels are essentially normal in IR48 *scar *null and IR55 *hspc300 *null cells. **(C) **Rescue of SCAR levels in IR55 *hspc300 *null cells expressing Myc-tagged or untagged HSPC300. Total lysates from wild type, *hspc300 *null and *hspc300 *null cells expressing either Myc-HSPC300 or an untagged control were blotted and probed with anti-SCAR antibodies. **(D) **HSPC300 is part of the SCAR complex. Lysates prepared from IR55 *hspc300 *null cells expressing either Myc-HSPC300 or an untagged control were immunoprecipitated with anti-Myc antibody and probed with anti-SCAR and anti-PIR121 antibodies.

The *Dictyostelium hspc300 *gene was first identified using a BLAST search against the *Dictyostelium *genome sequence using the human protein sequence as a bait [12]. This search identified Dictybase reference DDB0231424, which encodes a predicted protein of 68 amino acids and 8.9 kDa. *Dictyostelium *HSPC300, human HSPC300 and *Arabidopsis *BRICK1 are aligned in Figure 1A. The central 54aa of the *Dictyostelium *protein is 50% identical and 81% similar to human HSPC300, and 46% identical and 75% similar (using the BLOSUM62 matrix in each case) to *Arabidopsis *BRICK1. The human and *Arabidopsis *proteins have dissimilar N-termini, and the human HSPC300 has a significant, unconserved C-terminal extension. Like the other members of the *Dictyostelium *SCAR complex, HSPC300 is encoded by a single gene, and no other *Dictyostelium *genes were found to be significantly similar to known HSPC300 homologues [4-6].

### HSPC300 is required for SCAR protein stability

In a range of species, removal of one SCAR complex member (either genetically or by RNAi) leads to breakdown of others, apparently by posttranslational proteolysis [6,13]. In *Dictyostelium*, *pirA *and *napA *mutants contain barely detectable full length SCAR protein despite normal mRNA levels [5]. In the same fashion, disruption of *hspc300 *also leads to complete loss of SCAR- as shown in Figure 1B, western blots show no detectable SCAR protein. As seen in other mutants, SCAR mRNA levels in *hspc300 *null and wild type cells are comparable (data not shown), implying that the reduction in SCAR protein is a result of posttranslational processes such as degradation. However, PIR121 levels are insensitive to the loss of HSPC300 (Figure 1B). This insensitivity is also seen when *Dictyostelium *SCAR or Abi are lost [4,5], but loss of *Dictyostelium *Nap1 causes an approximate halving of PIR121 levels [5]. Taken together these results show that the mechanisms that regulate proteolytic breakdown affect different subunits of the complex independently. The PIR121 and Nap1 subunits appear to form a stable subcomplex that is preserved without SCAR, Abi or HSPC300 (see additional file 1), whereas SCAR is broken down in the absence of any other complex subunit.

### HSPC300 is a part of the SCAR complex

As predicted from other organisms *Dictyostelium *HSPC300 forms part of the complete SCAR complex. A previous *in vitro *study [14] showed that *in vitro *translated Abi and HSPC300 bind to the N-terminus of SCAR, but did not analyze the complex from living *Dictyostelium*. We expressed an N-terminally Myc-tagged HSPC300 or untagged control in *hspc300 *null cells from an extrachromosomal plasmid, which resulted in a restoration of normal SCAR protein levels (Figure 1C). In reciprocal co-immunoprecipitation experiments using an anti-Myc antibody, Myc-tagged HSPC300 coprecipitated with endogenous PIR121 and SCAR (Figure 1D). In other *Dictyostelium *SCAR complex mutants (except Nap1, in which PIR121 protein is lost), PIR121 is not recruited to the leading edge of actin protrusions as it is in wild type [4,5]. The *hspc300 *null cells also fail to recruit PIR121 to the edge of actin pseudopods (additional file 2), suggesting that the proposed Rac-binding site in PIR121 – which has not been verified in *Dictyostelium *– is not alone sufficient for localisation (data not shown).

### HSPC300 is needed for efficient cell motility

**Figure 2:**
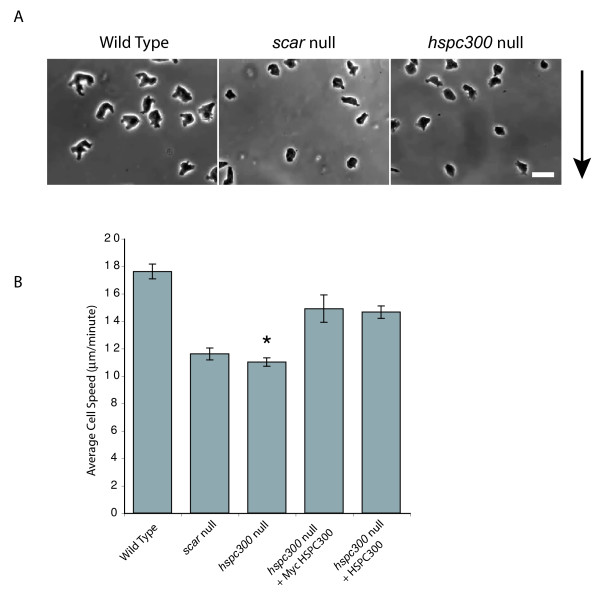
**HSPC300 is needed for efficient cell motility**. **(A) **Under-agar chemotaxis assay. Vegetative cells were allowed to migrate up a gradient of folate (arrow) under a layer of agar. Scale bar represents 20 μm. **(B) **Migration speed. Time-lapse movies made under conditions similar to (A) of 10 wild type, IR48 *scar *null, IR55 *hspc300 *null and *hspc300 *null expressing Myc-HSPC300 or untagged control cells were analyzed and a mean speed calculated. Data shown here are the means and standard errors of three independent experiments. Error bars represent one standard error. *hspc300 *null cells are significantly slower than wild type (P < 0.01) and are comparable to *scar *null cells. Expression of Myc-HSPC300 or an untagged control rescues the motility of *hspc300 *null cells back to wild type.

In some *Dictyostelium *SCAR complex mutants (particularly *pirA *and *abiA*), SCAR protein levels are diminished but the phenotype is not as severe as a complete *scar *disruption [4-6]. This suggests that some SCAR functions can be preserved in the absence of an intact complex. To determine if HSPC300 is needed for SCAR function we observed wild type (additional file 3), *scar *null (additional file 4) and *hspc300 *null (additional file 5) mutants migrating towards a stimulus, folate, under a thin layer of agar (Figure 2A) [15]. Wild type cells extended a number of large protrusions while migrating and move quickly in the direction of the stimulus. Cells lacking SCAR and HSPC300 both looked equally smaller and more rounded. These mutants can still initiate and protrude protrusions but these extensions are smaller compared to those extended by wild type cells. The speed of the mutant cells lines was measured from the movies using ImageJ and compared to the speed of wild type cells (Figure 2B). Cells lacking HSPC300 and SCAR are significantly slower than wild type cells, but not significantly different from one another. The expression of a Myc-tagged HSPC300 or an untagged control in *hspc300 *null cells restored wild type migration in the under-agar assay (Figure 2B). Similarly, cells lacking HSPC300 looked indistinguishable from cells lacking SCAR when F-actin was stained with phalloidin (additional file 6) – both contained less overall F-actin and fewer actin structures. The observations that F-actin, speed of migration and morphology of cells lacking either SCAR or HSPC300 are indistinguishable from one another suggest that the contribution of SCAR to migration has been entirely lost in *hspc300 *null cells.

### HSPC300 is not required for growth

**Figure 3:**
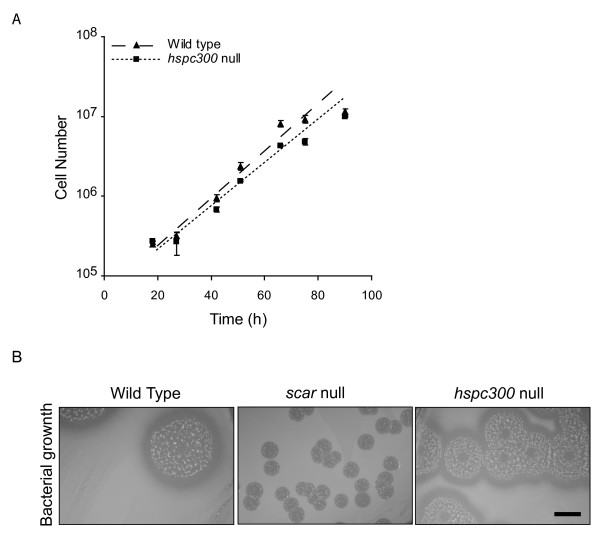
***hspc300 *null cell growth**. **(A) **IR55 *hspc300 *null cells do not have a growth defect in suspension. Cells were grown in shaken suspension and the cell density measured at regular intervals. Data shown here are the means and standard errors of three independent experiments. **(B) **Bacterial growth. Wild type, IR48 *scar *null and IR55 *hspc300 *null cells were plated on *Klebsiella aerogenes *lawns on SM agar plates. After 5 days of growth the colonies were photographed. Scale bar represents 1 cm.

SCAR is not needed for *Dictyostelium *growth, though it appears to aid efficient phagocytosis [16,17]. A difference is seen in *Arabidopsis *studies in which the control of pavement cell size is dependent on BRICK1 but not SCAR [10]. The authors suggest that this maybe due to HSPC300 having additional roles independent of SCAR. To determine if HSPC300 is important for any aspect of normal growth, growth curves were plotted for wild type and *hspc300 *null cells (Figure 3A). *hspc300 *null cells have a population doubling time of approximately 13 hours. This is very similar to the estimated population doubling time of wild type cells. Somewhat unexpectedly, when grown on *Klebsiella *lawns, *hspc300 *null colonies are only slightly smaller than wild type, and retain the wild type morphology with a defined leading edge (Figure 3B). *scar *null colonies are extremely small and have an extremely narrow feeding front. We therefore find no evidence that HSPC300 has functions outside of the SCAR complex, but the less severe phenotype on lawns could perhaps result from a small amount of residual SCAR activity in the absence of HSPC300. This seems similar to *Dictyostelium *cells lacking Abi, in which residual SCAR activity is responsible for the phenotype [4].

### HSPC300 and development

**Figure 4:**
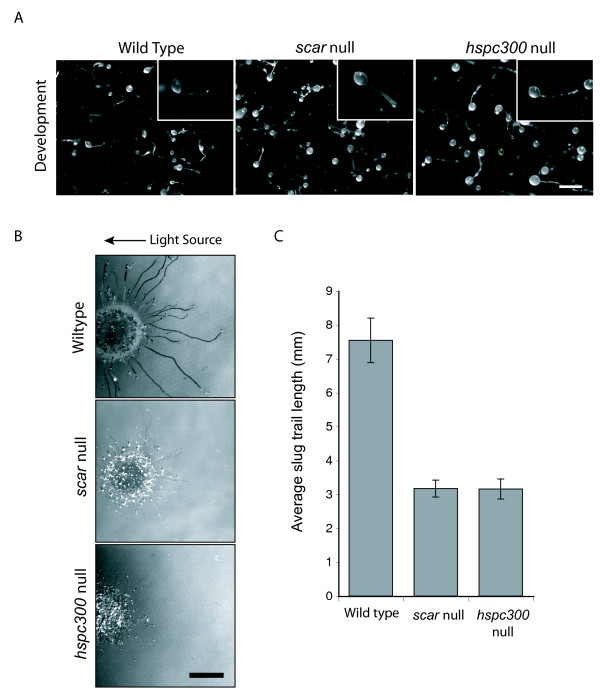
**The role of HSPC300 in development**. **(A) **IR55 *hspc300 *null cells have no developmental defects. Wild type, IR48 *scar *null and *hspc300 *null cells were developed on nitrocellulose filters for 24 hours. The scale bar represents 0.5 mm. **(B) **Phototaxis of *hspc300 *null cells. Wild type, *scar *null and *hspc300 *null cells were allowed to form slugs and migrate towards a unidirectional light source, indicated by the arrow. Scale bar represents 0.5 cm. **(C) **Migration distance of wild type, *scar *null, *hspc300 *null slugs. Error bars represent one standard error. *scar *null and *hspc300 *null cells are significantly slower than wild type (P < 0.01).

Neither *scar *nor *hspc300 *null cells have obvious developmental defects (Figure 4A). When developed on filters these mutants form morphologically normal fruiting bodies which cannot be easily distinguished from wild type fruiting bodies. This is surprising considering that multiple aspects of cell movement, adhesion and contractility are required for multicellular development, and that in other organisms the loss of SCAR leads to severe developmental defects [18,19]. Developed cells moving towards cAMP behave similarly to vegetative NC4A2 moving towards folate (additional files 7 &8) – the cells are slower and pseudopods are smaller, but movement and chemotaxis are remarkably robust. We believe that aggregating *Dictyostelium *cells, like many others, have multiple mechanisms to drive migration. One clear candidate is based on protrusion of blebs [20], and there may be others. Mutants in members of the SCAR complex can presumably aggregate using these.

As development was not greatly affected by the loss of HSPC300 we investigated slug behaviour in the *hspc300 *null cells to see if there were any subtle differences during this stage of development. Wild type slugs will phototax effectively towards a single point source of light when allowed to develop in the dark (Figure 4B). *hspc300 *and *scar *null slugs were still capable of migrating towards the light source but not to the same extent as wild type slugs. The lengths of the tracks trailing the slugs were analysed using ImageJ (Figure 4C). Although significantly shorter than wild type slug trails, *hspc300 *and *scar *null tracks were found to be very similar in length. Thus during phototaxis, as in most other conditions, HSPC300 is required for SCAR function as the loss of HSPC300 results in a *scar *null phenotype.

## Conclusion

We have demonstrated that HSPC300 is a vital component of the *Dictyostelium *SCAR complex. Unlike *Dictyostelium *Abi and PIR121, HSPC300 seems to be fully required for most SCAR functions, though phagocytosis may be better in *hspc300 *than in *scar *knockouts, raising the possibility that SCAR's role in phagocytosis does not require HSPC300. We found no evidence that HSPC300 has roles outside the SCAR complex, as has been suggested in *Arabidopsis*. Although this small protein is vital for the stability of the SCAR complex it remains unclear what its physiological role may be, and why such a small protein is required physiologically if its only role is as a partner for SCAR.

## Methods

### Cell Culture and Development

*Dictyostelium *cells were grown axenically in HL-5 medium at 22°C in Petri dishes. For growth curves, cells were grown axenically in shaken flasks. For development on filters 10^7 ^axenically grown cells were washed in KK_2 _(16 mM potassium phosphate pH6.2) buffer and evenly distributed onto nitrocellulose filters (45 μm, Black HABP, Millipore), placed on KK_2 _soaked filter pads (absorbent pads, Millipore). For cloning on bacteria, serial dilutions of axenically grown *Dictyostelium *cells were spread onto *Klebsiella aerogenes *lawns on SM agar plates and incubated for 5 days to gain single colonies.

### Generation of Gene Disruptants

The *Dictyostelium discoideum *homologue of HSPC300 was identified by BLAST searches against the *Dictyostelium *genome , using the human protein as bait. A 1.69 Kb section of the *hspc300 *gene and flanking DNA was amplified from genomic DNA by PCR, with a BamHI site introduced by 4-primer mutagenesis. A blasticidin resistance cassette derived from pBsr∂Bam was cloned into the BamHI site introduced into the *hspc300 *gene. This construct was electroporated into *Dictyostelium *AX3 and NC4A2 cells. NC4A2 is a line which migrates effectively and in which SCAR shows consistent phenotypes in vegetative and developed cells, originally published as being independently axenised from NC4 [21]; subsequent work suggests that it is an AX3 contaminant [22] though we have not yet squared this with its phenotypic difference from other AX3 strains. After cloning on bacterial lawns, blasticidin resistant colonies were screened for gene disruption by PCR (see additional file 9; Forward primer: ATCTTTTTGGTGTAATCATTGGTG, Reverse Primer: TAGATCAAGAAAAACTTAATGATCG), looking for replacement of the original small fragment by one 1.3 kbp larger. Two AX3 and one NC4A2 lines were isolated, with similar phenotypes. The NC4A2-derived line (IR55) was used for the work described in this paper, with the AX3-derived one (IR54) also kept for comparison.

### Phototaxis Assay

10^7 ^washed axenically grown cells were concentrated in 50 μl KK_2 _buffer. Cells were placed as a droplet onto a damp nitrocellulose filter placed on KK_2 _soaked filter pads. Excess buffer was removed and slugs allowed to migrate towards a unidirectional light source. Images were captured after 24 hours. The length of the slug trails were analysed using ImageJ.

### Immunoprecipitation of Myc tagged HSPC300

Extrachromosomal constructs containing either an N-terminal Myc-tagged HSPC300 or untagged HSPC300 control under the control of an Actin15 promoter were generated and electroporated into *hspc300 *null cells. Whole cell lysates were made in a Triton containing buffer (50 mM Tris-HCL pH7, 150 mM NaCl, 1% Triton, 1 mM EDTA). Pre-cleared lysates were subjected to immunoprecipitation with mouse monoclonal anti-Myc antibodies (9E10) for 30 mins at 4°C and captured using protein-G-coupled sepharose beads for 1 hour at 4°C. Beads were washed four times with lysis buffer and resuspended in SDS-PAGE sample buffer. Proteins were resolved on a SDS-PAGE gel and PIR121 and SCAR detected using sheep anti-PIR121 and sheep anti-SCAR antibodies [5,23].

### Blue Native PAGE

Cells were washed in KK_2_, then protein from 2 × 10^5 ^separated using Invitrogen NativePAGE™ Novex blue gels. The cells were lysed using the kit lysis buffer, centrifuged briefly in a desktop centrifuge, then run on 3–12% gradient gels using the recommended voltage and kit buffers. After separation gels were transferred to nitrocellulose then treated the same way as SDS-PAGE immunoblots.

### Under Agar Chemotaxis Assays and Microscopy

We used the previously described method for imaging *Dictyostelium *cells moving towards a folate stimulus under a thin layer of agar [15]. Time-lapse phase images were generated with frames captured every 10 seconds. Cell speeds were calculated using ImageJ.

For phalloidin staining and immunofluorescence, cells were fixed and permeabilised with picrate/formaldehyde then stained with Texas red-conjugated phalloidin as described elsewhere [4].

## Authors' contributions

AP performed all the experiments, jointly conceived the work, and jointly wrote the paper. RI jointly conceived the work, and jointly wrote the paper. Both authors have read and approved the final manuscript.

## Supplementary Material

Additional file 1 **PIR121 and SCAR subcomplexes in mutants**. Vegetative wild type and mutant cells were lysed and intact protein complexes were separated using blue native PAGE. Gels were blotted onto Nitrocellulose then probed with anti-PIR121 (top) and anti-SCAR (bottom) antibodies. Subcomplex 1, which was seen in all mutants examined except *pirA *nulls, is only slightly smaller than the intact complex and thus presumably represents a complex of the Nap1 and PIR121 subunits. Subcomplex 2 is only seen in *abiA *null cells and may represent either unbound SCAR or (more likely) SCAR+HSPC300. Click here for file

Additional file 2 **PIR121 localisation in *hspc300*^- ^cells**. Vegetative wild type and IR55 *hspc300*^- ^cells were fixed using picrate/formaldehyde and stained using an anti-PIR121 antibody (left panels, green in right panels) and phalloidin (red in right panels). PIR121 is found at the extreme leading edge of wild type cells but never in *hspc300*^- ^mutants. Scale bar is 2 μM. Click here for file

Additional file 3 **Under agar folate chemotaxis of wild type cells**. Movie of wild type cells moving towards a folate gradient under agar. Cells were imaged using phase contrast microscopy. Frames were taken every 30 seconds and are played at 10 frames/second. Click here for file

Additional file 4 **Under agar folate chemotaxis of *scar *null mutant**. Movie of *scar *null cells moving towards a folate gradient under agar. Cells were imaged using phase contrast microscopy. Frames were taken every 30 seconds and are played at 10 frames/second. Click here for file

Additional file 5 **Under agar folate chemotaxis of *hspc300 *null mutant**. Movie of *hspc300 *null cells moving towards a folate gradient under agar. Cells were imaged using phase contrast microscopy. Frames were taken every 30 seconds and are played at 10 frames/second. Click here for file

Additional file 6 **F-actin in *hspc300 *mutants**. Vegetative wild type NC4A2, IR55 *hspc300*^- ^and IR46 *scar*^- ^cells were fixed using picrate/formaldehyde, and F-actin was stained using Texas red-conjugated phalloidin. Wide-field images were taken using a Zeiss Axiovert 100 with a 63×, NA 1.4 achroplan objective. All three samples were handled and imaged under identical conditions. Scale bar represents 10 μm. Click here for file

Additional file 7 **Under agar cAMP chemotaxis of wild type cells**. Movie showing wild type cells moving up a cAMP gradient under agar as described in [24]. Cells were imaged using DIC microscopy. Frames were taken every 30 seconds and are played at 10 frames/second. Click here for file

Additional file 8 **Under agar cAMP chemotaxis of *hspc300 *null mutants**. Movie showing *hspc300 *null cells moving up a cAMP gradient under agar as described in [24]. Cells were imaged using DIC microscopy. Frames were taken every 30 seconds and are played at 10 frames/second. Click here for file

Additional file 9 **PCR confirmation of *hspc300 *disruption**. Vegetative wild type NC4A2 and IR55 *hspc300*^- ^cells were screened by PCR using primers upstream and downstream of the knockout construct as described in Materials & Methods. The wild type band is 1.7 kbp long and the *hspc300 *gene disruption band is 3 kbp long (1.7 kbp and 1.3 kbp for the blasticidin insert). Click here for file
