# Supplementary figures and images for: Loss of Dictyostelium HSPC300 causes a scar-like phenotype and loss of SCAR protein
Source: BMC Cell Biol. 2009 Feb 19;10:13. doi: 10.1186/1471-2121-10-13 (PMC2652429; doi:10.1186/1471-2121-10-13)

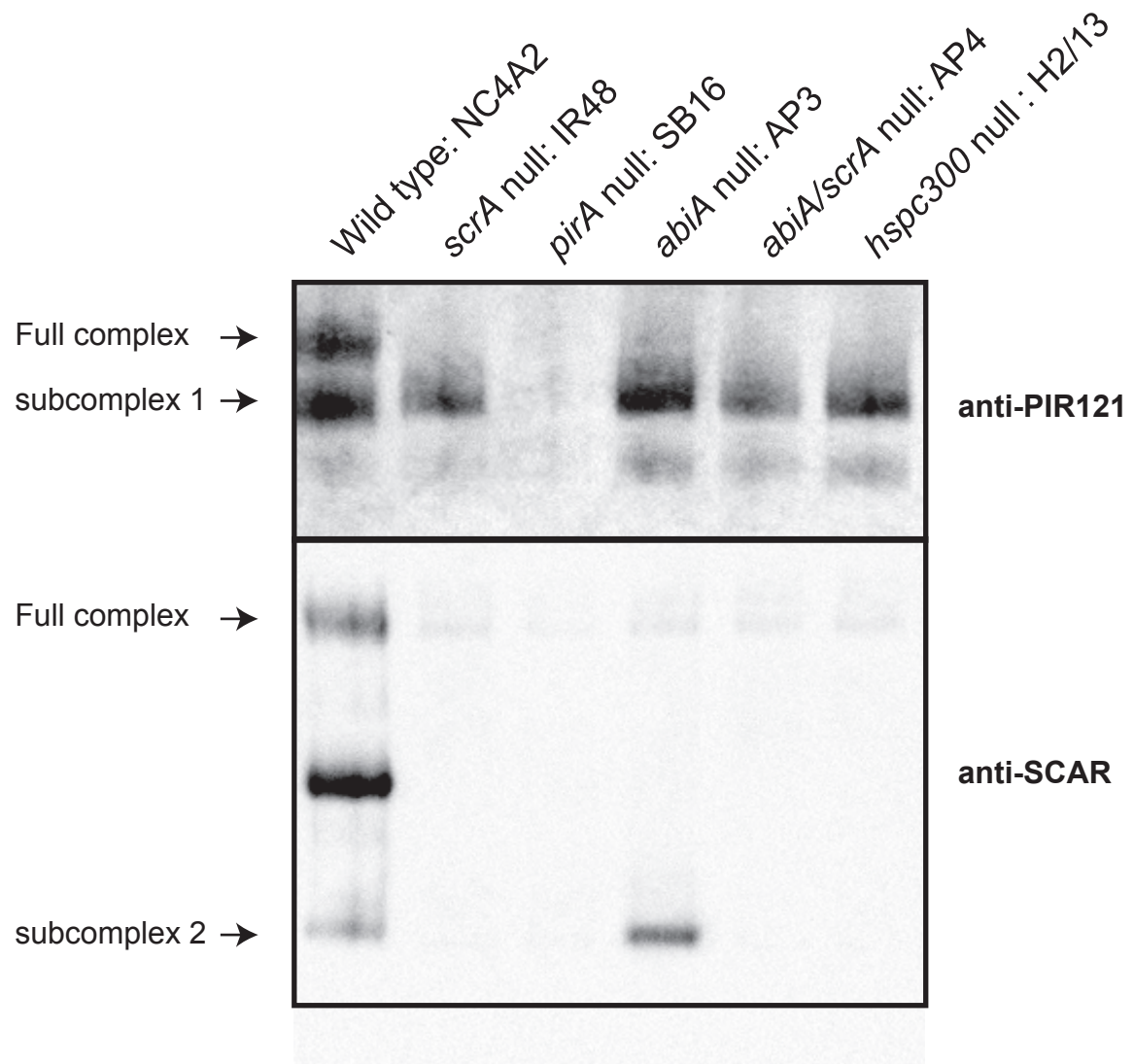

Supplement: Additional file 1 — PIR121 and SCAR subcomplexes in mutants. Vegetative wild type and mutant cells were lysed and intact protein complexes were separated using blue native PAGE. Gels were blotted onto Nitrocellulose then probed with anti-PIR121 (top) and anti-SCAR (bottom) antibodies. Subcomplex 1, which was seen in all mutants examined except pirA nulls, is only slightly smaller than the intact complex and thus presumably represents a complex of the Nap1 and PIR121 subunits. Subcomplex 2 is only seen in abiA null cells and may represent either unbound SCAR or (more likely) SCAR+HSPC300. [file 1471-2121-10-13-S1.pdf]

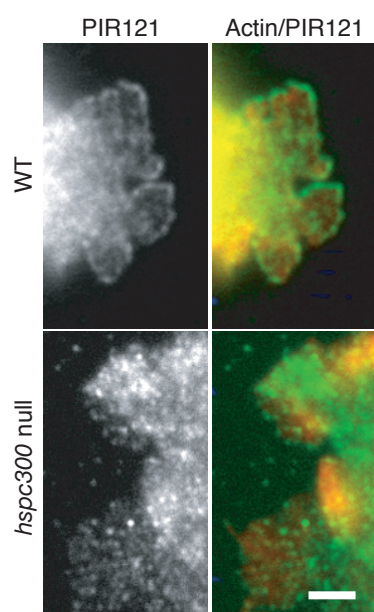

Supplement: Additional file 2 — PIR121 localisation in hspc300- cells. Vegetative wild type and IR55 hspc300- cells were fixed using picrate/formaldehyde and stained using an anti-PIR121 antibody (left panels, green in right panels) and phalloidin (red in right panels). PIR121 is found at the extreme leading edge of wild type cells but never in hspc300- mutants. Scale bar is 2 μM. [file 1471-2121-10-13-S2.pdf]

Wild Type

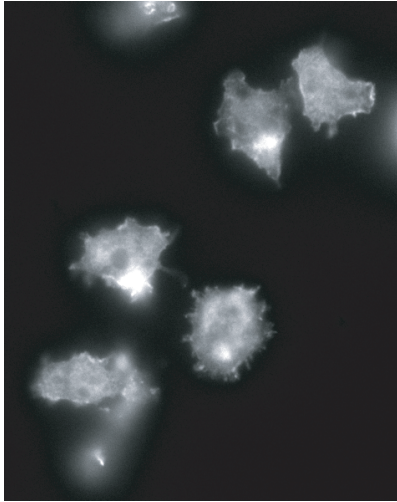

*hspc300* null

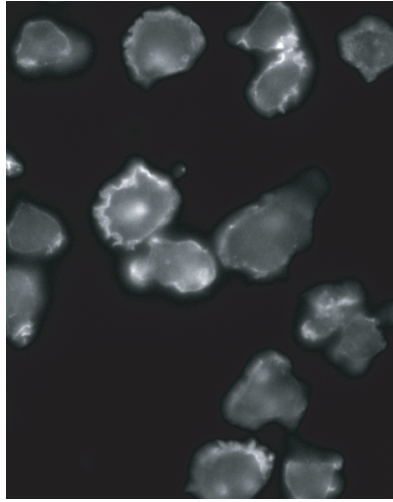

*scar* null

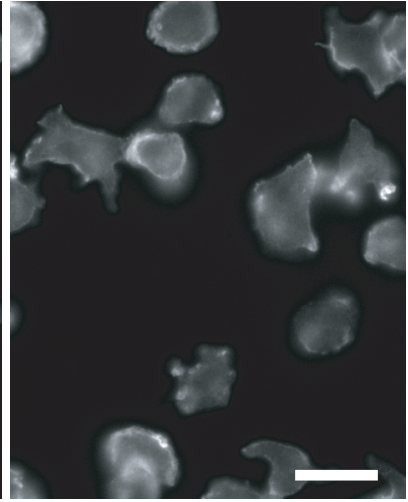

Supplement: Additional file 6 — F-actin in hspc300 mutants. Vegetative wild type NC4A2, IR55 hspc300- and IR46 scar- cells were fixed using picrate/formaldehyde, and F-actin was stained using Texas red-conjugated phalloidin. Wide-field images were taken using a Zeiss Axiovert 100 with a 63×, NA 1.4 achroplan objective. All three samples were handled and imaged under identical conditions. Scale bar represents 10 μm. [file 1471-2121-10-13-S6.pdf]

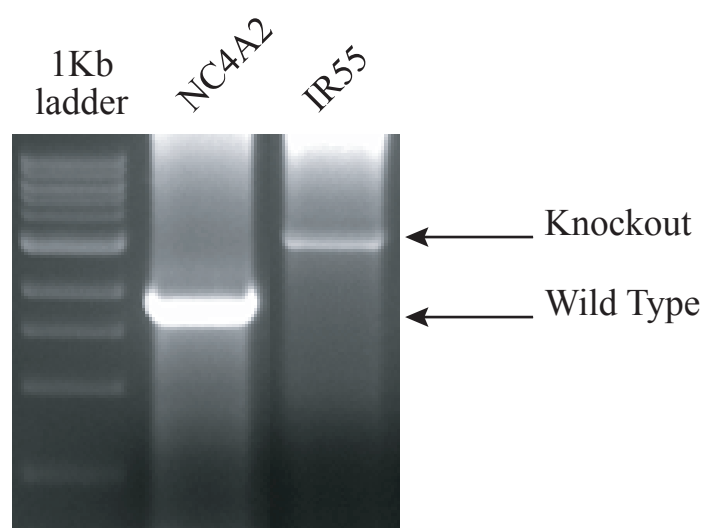

Supplement: Additional file 9 — PCR confirmation of hspc300 disruption. Vegetative wild type NC4A2 and IR55 hspc300- cells were screened by PCR using primers upstream and downstream of the knockout construct as described in Materials & Methods. The wild type band is 1.7 kbp long and the hspc300 gene disruption band is 3 kbp long (1.7 kbp and 1.3 kbp for the blasticidin insert). [file 1471-2121-10-13-S9.pdf]
